# UBA5 Mutations Cause a New Form of Autosomal Recessive Cerebellar Ataxia

**DOI:** 10.1371/journal.pone.0149039

**Published:** 2016-02-12

**Authors:** Ranhui Duan, Yuting Shi, Li Yu, Gehan Zhang, Jia Li, Yunting Lin, Jifeng Guo, Junling Wang, Lu Shen, Hong Jiang, Guanghui Wang, Beisha Tang

**Affiliations:** 1 Department of Neurology, Xiangya Hospital, Central South University, Changsha, Hunan province, China; 2 The State Key Laboratory of Medical Genetics, School of Life Sciences, Central South University, Changsha, Hunan province, China; 3 Department of Pharmacology, College of Pharmaceutical Sciences, Soochow University, Suzhou, Jiangsu province, China; 4 The Key Laboratory of Hunan Province in Neurodegenerative Disorders, Central South University, Changsha, Hunan province, China; Emory University, UNITED STATES

## Abstract

Autosomal recessive cerebellar ataxia (ARCA) comprises a large and heterogeneous group of neurodegenerative disorders. For many affected patients, the genetic cause remains undetermined. Through whole-exome sequencing, we identified compound heterozygous mutations in ubiquitin-like modifier activating enzyme 5 gene (*UBA5*) in two Chinese siblings presenting with ARCA. Moreover, copy number variations in *UBA5* or ubiquitin-fold modifier 1 gene (*UFM1*) were documented with the phenotypes of global developmental delays and gait disturbances in the ClinVar database. *UBA5* encodes UBA5, the ubiquitin-activating enzyme of UFM1. However, a crucial role for UBA5 in human neurological disease remains to be reported. Our molecular study of UBA5-R246X revealed a dramatically decreased half-life and loss of UFM1 activation due to the absence of the catalytic cysteine Cys250. UBA5-K310E maintained its interaction with UFM1, although with less stability, which may affect the ability of this UBA5 mutant to activate UFM1. Drosophila modeling revealed that UBA5 knockdown induced locomotive defects and a shortened lifespan accompanied by aberrant neuromuscular junctions (NMJs). Strikingly, we found that UFM1 and E2 cofactor knockdown induced markedly similar phenotypes. Wild-type UBA5, but not mutant UBA5, significantly restored neural lesions caused by the absence of UBA5. The finding of a *UBA5* mutation in cerebellar ataxia suggests that impairment of the UFM1 pathway may contribute to the neurological phenotypes of ARCA.

## Introduction

Autosomal recessive cerebellar ataxias (ARCAs) are a large group of neurodegenerative disorders that manifest mainly in children and young adults. Most ARCAs are heterogeneous with respect to the age at onset, severity of the disease progression, and frequency of extracerebellar and systemic signs. [1,2] Five main pathogenic mechanisms are distinguishable: defective DNA repair, abnormal protein folding and degradation, channelopathies, and mitochondrial and metabolic defects. [3] Although a growing list of rare molecular defects associated with ARCAs have been identified, as yet many affected families and individuals have an unknown etiology. [4,5,6]

We applied whole-exome sequencing to DNA from two siblings with progressive cerebellar ataxia who had non-consanguineous parents. Consequently, compound heterozygous variants were found in *UBA5*. UBA5, also known as ubiquitin-like modifier activating enzyme 5, is an ubiquitin-activating enzyme (E1) of the ubiquitin-fold modifier 1 (UFM1) pathway. UFM1 is an ubiquitin-like protein (UBL) that, upon activation by a dedicated E1 (UBA5), forms a thioester bond with an E2 cofactor (UFC1), resulting in the tagging of reactive ubiquityl units to substrates by an E3 ligase (UFL1).[7–11] In ischemic myocardial cells and pancreatic islet beta cells, endoplasmic reticulum (ER) stress can specifically induce the expression of UFM1 cascade members, thus suggesting the protection of the UFM1 cascade during cellular homeostasis.[12,13] In UBA5, the catalytic cysteine (Cys250) is part of the adenylation domain within the helical motif through which the ubiquityl-enzyme thioester is formed.[14] Our cellular studies revealed that both of the two mutants (p.R246X and p.K310E) became less stable which caused reduced enzymatic activity of UBA5. A role for UBA5 in human neurological disease has yet to be identified. Here we studied a cohort of patients with ARCA and identified *UBA5* mutations in these patients.

*Drosophila* is a powerful model organism with which to study neural development and neuronal maintenance. A *Drosophila* model containing a loss-of-function of the UBA5 homologue confirmed the role of this in disease pathogenesis. In particular, we found that UBA5 knockdown resulted in remarkably reduced locomotor activity, a shortened lifespan, and neuromuscular junction (NMJ) defects. Furthermore, similar phenotypes were observed with UFM1 and UFC1 knockdown, although UFM1 knockdown resulted in a more severe phenotype. *Drosophila* NMJs provide a simpler and genetically more amenable system with which to explore the molecular mechanisms underlying synapse development and maturation.[15] Both *Drosophila* and human wild type UBA5, but not UBA5 mutations, significantly rescue the neuromuscular junction (NMJ) defects. Our data therefore establish a novel ARCA syndrome by a mutation in *UBA5* and thus shed light on the biological function of the corresponding protein.

## Materials and Methods

### Ethic statement

This study protocol was approved by the Ethic Committee of the Xiangya Hospital of Central South University in China (equivalent to an Institutional Review Board). The individual in this manuscript have given written informed consent.

### Patients

Clinical data and blood samples were obtained from two Chinese siblings who presented with progressive ataxia during childhood and had non-consanguineous parents. Both patients underwent a standardized neurologic examination conducted by two neurological specialists. We used DNA sequencing and capillary electrophoresis to exclude mutations and repeat expansions in known ataxia genes. In addition, 500 unaffected, healthy Chinese individuals were analyzed as controls. The relevant ethical authorities approved this study, and written informed consent was obtained from all subjects.

### Whole-exome sequencing

Genomic DNA was extracted from the peripheral blood of the two affected individuals (II:2 and II:3) using standard methods (QIAGEN, Valencia, CA). Whole-exome sequencing was performed using a Genome Analyser II platform. Sequencing data were aligned to the human genome reference (UCSC hg 18 version). The variants were confirmed using Sanger sequencing.

### Plasmid construction, cell culture, and transfection

Human wild-type UBA5 cDNA was PCR amplified from a human cDNA library and inserted in-frame into p3xFlag-CMV-24 (Sigma, St. Louis, MO, USA) at the EcoRI/SalI sites. Mutants were generated via QuikChange site-directed mutagenesis according to the manufacturer’s protocol (Stratagene, La Jolla, CA, USA). All constructs were confirmed by sequencing. Human embryonic kidney 293A cells (HEK293A, Invitrogen, R705-07) and human cervical carcinoma HeLa cells (Chinese Academy of Sciences, TCHu187) were cultured in Dulbecco’s Modified Eagle Medium (Invitrogen, Carlsbad, CA, USA) with 10% fetal bovine serum (FBS) at 37°C and 5% CO_2_. Plasmids were transfected into cells using Lipofectamine 2000 (Invitrogen).

### Degradation assay, immunoprecipitation, and westernblotting

After transfection, HEK293A cells expressing the indicated plasmids were treated with 100 μg/ml cycloheximide (CHX; Sigma). The cells were harvested after 0, 12, 24, and 36 h of CHX treatment. For co-immunoprecipitation, the following antibodies were used: monoclonal anti-Flag (Sigma); monoclonal anti-UFM1 (Epitomics/Abcam, USA); monoclonal anti-GAPDH (Sigma); polyclonal anti-UBA5 (Epitomics/Abcam), and sheep anti-rabbit antibody (Amersham Pharmacia Biotech).

### Immunocytochemical analyses

Cells transfected with the indicated plasmids were grown on cover slides and fixed with 4% paraformaldehyde (PFA) for 5 min at room temperature; the cells were subsequently incubated with 0.25% Triton X-100 for 5 min and blocked with 0.1% FBS in phosphate-buffered saline (PBS). DAPI (Sigma) was used for nuclear staining. Anti-UBA5 (Epitomics/Abcam) and anti-GM130 antibodies (Sigma) were also used for staining. All cells were imaged using a fluorescence microscope equipped with a cooled charge-coupled device camera (CTR MIC; Leica, Wetzlar, Germany).

Third-instar larval muscles were dissected and stained using a modified protocol described by Dr. Wei Xie.[16] Larval muscles were fixed in 4% PFA and incubated with anti-DLG antibody (DSHB) at a dilution of 1:50. The NMJs from muscle 4 of abdominal segments 2, 3, and 4 were imaged using a Leica TCS SP5 confocal station and ImageJ software (National Institutes of Health, Bethesda, MD, USA) to quantify the bouton numbers and sizes.

### *Drosophila* genetics and fly stocks

Fly cultures and crosses were performed according to standard procedures. The *DA-GAL4*, *UBA5 RNAi*, *UFM1 RNAi*, *w*^*1118*^ and *attP2* fly lines were obtained from the Bloomington *Drosophila* Stock Center at Indiana University (Bloomington, IN, USA). *UFC1 RNAi* and *UFL1 RNAi* fly lines were obtained from the Vienna *Drosophila* RNAi Center (Vienna, Austria). The *OK6-GAL4* fly line was a gift from Dr. Wei Xie at Southeast University. Wild-type UBA5 cDNA was amplified from *Drosophila* cDNA. Human wild-type and mutant UBA5 cDNA sequences were HA-tagged and subcloned into the pUAST vector at the EcoRI/XhoI restriction sites. P-element–mediated germ line transformations were performed via microinjection into *w*^*1118*^ background flies using P-element vectors (pUAST-HA-dUBA5-WT, pUAST-HA-hUBA5-WT, pUAST-HA-hUBA5-R246X, and pUAST-HA-hUBA5-K310E). F1 transformants were selected on the basis of white eye-color rescue.

### Quantification of wing phenotypes and light microscopy

The percentage of 3-day-old male flies that exhibited unfolded vertical-turned wings was measured (n ≥ 300). Whole flies were analyzed using an OLYMPUS DP72 microscope (Olympus Corporation, Tokyo, Japan) to obtain light microscopy (LM) images.

### Behavioral and longevity assays

For climbing assays, groups of ten 3-day-old male flies were transferred into 1.25-cm-diameter and 28-cm-height plastic tubes for a 30-min incubation at room temperature. The time at which the fifth fly arrived at the 15-cm finish line was recorded and analyzed. For flight assays, a vial of twenty 3-day-old male flies was guided into a 500-ml measuring cylinder. In this test, normal flies run into the inner wall of the measuring cylinder and are glued to the top, whereas disabled flies fall straight to the bottom. For longevity assays, 20 male flies were placed in a vial maintained at 25°C and supplied with fresh food every 3 days. Five vials were recorded per genotype. The above assays were repeated at least 3 times per genotype.

## Results

### Clinical description of the UBA5 family

**Fig 1:**
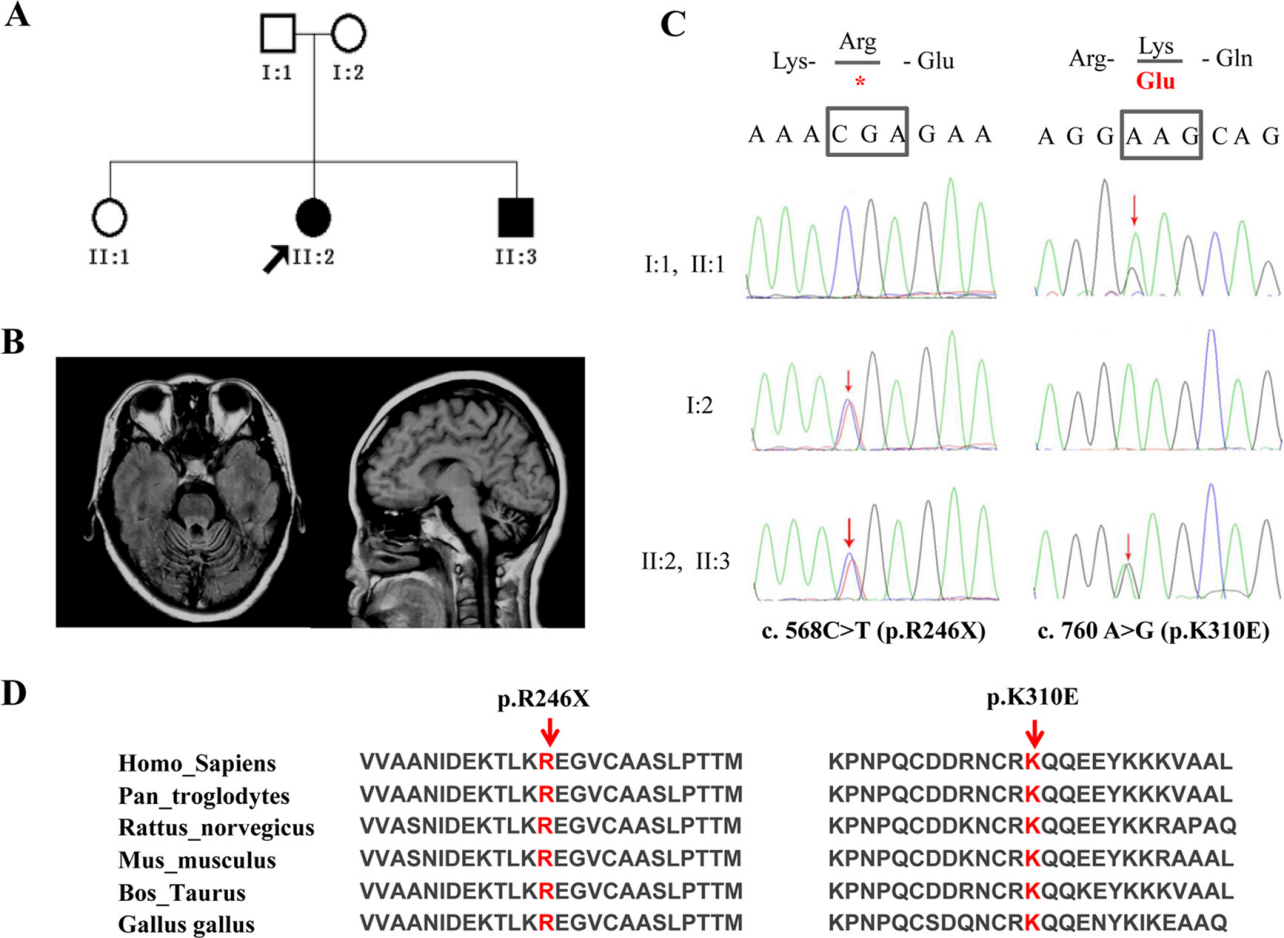
Genetic findings in a family with *UBA5* mutations. (A) The pedigree of family 1 with autosomal recessive spinocerebellar ataxia. (B) Brain magnetic resonance imaging of patient II:2. Panel (left): axial T1-weighted image showing atrophy of the cerebellar vermis. Panel (right): midline sagittal T1-weighted image showing cerebellar atrophy, particularly in the superior vermis, with enlargement of the fourth ventricle. (C) The UBA5 variants [c. 568C > T; c. 760A > G], [p. Arg246X (R246X); p. Lys310Glu (K310E)] segregated in this family. Red arrows indicate the mutation sites. (D) Two mutations (red) affected amino acids that are highly conserved across species.

The affected family originated from the Shanxi province in China (Fig 1A). The parents (I:1 and I:2) were non-consanguineous. The father of the affected siblings (II:2 and II:3) reported no gait difficulties at the age of 80, and the mother presented with an essential tremor of the head but no gait difficulties. Neurologic examinations of the parents (I:1 and I:2) yielded normal findings. The two affected siblings, one female and one male, reported gait instability since childhood. Both siblings exhibited markedly delayed growth during childhood but had achieved a normal body size in adulthood. Both developed cataracts while young and had undergone cataract-removal surgeries in 2010. Neither sibling exhibited signs of cognitive involvement. The disease progressed insidiously in the proband (II:2), who presented with marked cerebellar atrophy as determined by brain magnetic resonance imaging (MRI; Fig 1B), leading to a loss of the ability to walk and caregiver dependency at 39 years of age. The disease course was apparently stable in the younger brother (II:3), who was 36 years old, worked full-time, and displayed a mildly spastic gait that did not interfere with his daily activities, and also exhibited mild cerebellar atrophy on MRI. Neurophysiologic studies indicated no other central or peripheral system impairments in the proband. The brother (II:3) exhibited partial peripheral nerve impairment with a demyelinating sensory-motor peripheral neuropathy. Phenotypic details are given in Table 1.

**Table 1:** The phenotypic characteristics of patients with intermediate.

	Patient II:2	Patient II:3
**Age (y)**	40	36
**Sex**	Female	Male
**Age at onset (y)**	5	8
**Symptoms**	Gait instability, speech difficulties	Gait instability, speech difficulties
**Clinical signs**	Gait and limb ataxia, dysarthria, horizontal nystagmus	Gait and limb ataxia, dysarthria, horizontal nystagmus
**Other symptoms**	Cataract	Cataract
**EMG and NCV**	Normal	EMG: Normal; NCV: partial abnormality
**Evoked potentials**	VEP: normal; AEP: normal;SEP: increased latency in RLL	VEP: normal; AEP: increased latency; SEP: normal
**ICARS**	46	29
**SARA**	17	11
**MMSE**	27	30
**Brain MRI**	Cerebellar atrophy	Cerebellar atrophy

### Whole-exome sequencing identified UBA5 mutations in siblings with ARCA

The non-consanguineous pedigree of the index family includes two siblings (patient II:2 and II:3) with ataxia. DNA from both affected individuals was subjected to whole-exome sequencing. Variants were prioritized according to the presence of compound heterozygosity and homozygosity, based on recessive inheritance. Only two variants in the *UBA5* (NM_198329.2) cosegregated completely in the conserved domain of the UBA5 protein: c.568C > T; p. R246X and c.760A > G; p. K310E (Fig 1C and 1D). These variants were not identified in any of the 500 unaffected controls.

### Characterization of UBA5 and its mutant protein

**Fig 2:**
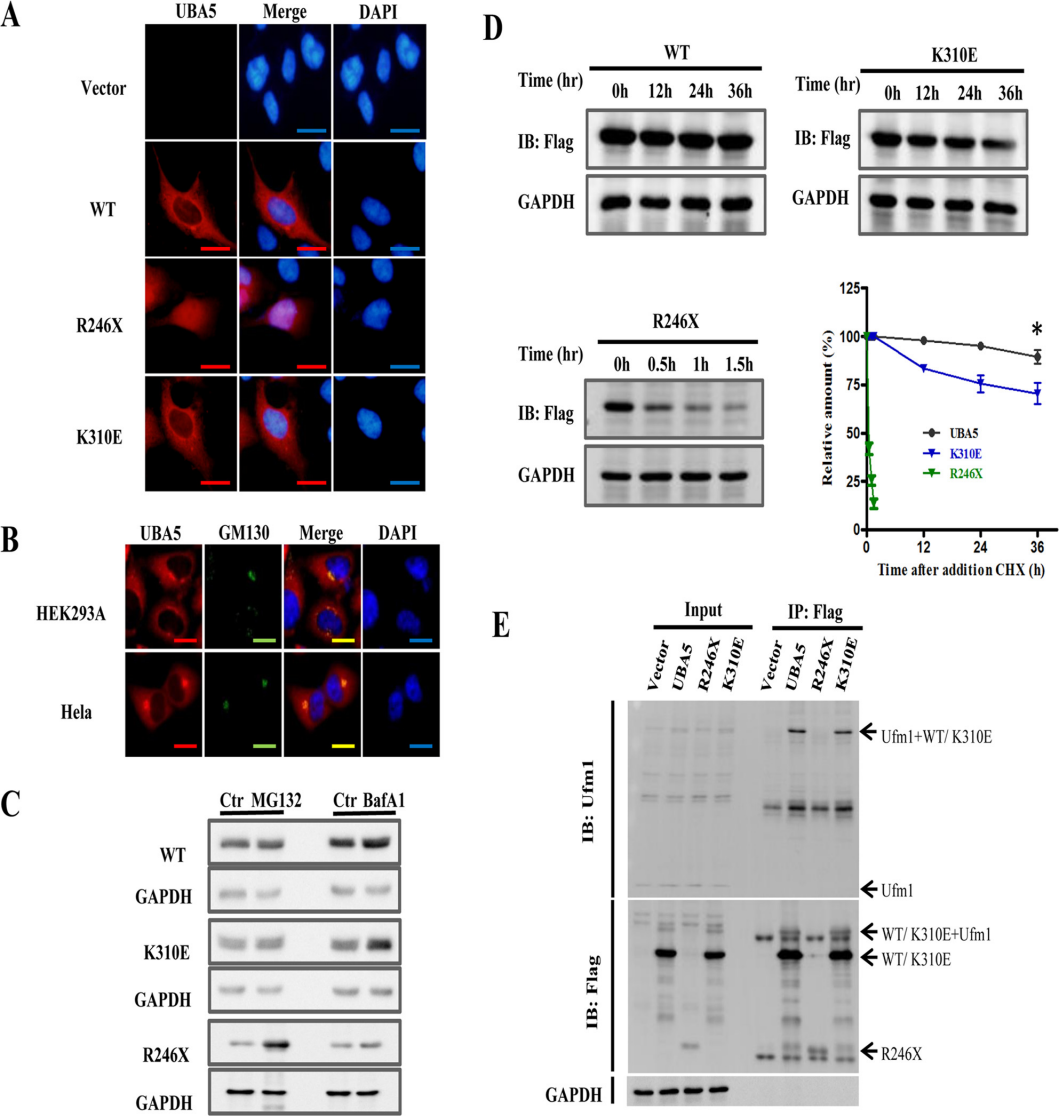
The subcellular localization, manner of degradation, and stability of mutant UBA5 and its interaction with UFM1. (A) Immunostaining of overexpressed UBA5 and its mutants, as well as endogenous UBA5. (B) Immunostaining of endogenous UBA5 in HEK293A and HeLa cells. (C) Flag-UBA5 or related mutants were overexpressed via plasmid transfection in HEK293A cells. After 24 h, the cells were treated with MG132 (100μg/ml) or Bafilomycin A1 (BafAl, 100μg/ml). (D) UBA5 stability analysis. (E) Interactions of UBA5 mutants with Ufm1.

Immunofluorescence was used to visualize exogenously expressed WT and mutant UBA5 in HEK293A cells. As shown in Fig 2A, wild-type and K310E UBA5 were localized in the cytoplasm, whereas the truncated R246X protein was mainly localized in the nucleus. We also found that endogenous UBA5 co-localized with GM130 (Golgi marker) (Fig 2B). We next aimed to investigate the degradation of UBA5. After overexpressing UBA5 in HEK293A cells, MG132 or bafilomycin A1 (BafAl) was added to the cells, followed by a 12-h incubation. The results demonstrated a marked increase in UBA5 protein levels after the addition of BafA1 (autophagy inhibitor), but not MG132 (proteasome inhibitor), indicating that UBA5 was degraded through autophagy. K310E protein was degraded in the same manner as wild-type UBA5 (Fig 2C). However, levels of the truncated R246X protein increased significantly in the presence of MG132 but not BafA1, suggesting that the R246X protein was degraded through the ubiquitin proteasome pathway (Fig 2C). To examine whether *UBA5* mutations impaired the stability of the protein product, a CHX-chase analysis was performed. The results indicated that wild-type UBA5 protein was quite stable with a half-life of more than 36 h, whereas K310E was less stable and R246X protein had a half-life of less than 30 min (Fig 2D).

### Activity of UBA5 mutants with regard to UFM1

UBA5 is among the least characterized of all human E1 enzymes. In a previous report, UBA5 was shown to activate UFM1 and SUMO2. [8] However, we and others demonstrated a lack of interaction between UBA5 and SUMO2 (data not shown). Next, we focused on UFM1. A co-immunoprecipitation study demonstrated that R246X protein failed to interact with endogenous UFM1, indicating that this mutation impaired the ability of UBA5 to activate UFM1 (Fig 2E). K310E remained able to interact with UFM1, similar to wild-type UBA5; thus, the pathogenesis associated with this mutation will require further investigation (Fig 2E).

### Impairment of the UFM1 pathway causes an abnormal *Drosophila* phenotype

*Drosophila* UBA5 (dUBA5, CG1749) is highly homologous to human UBA5 and shares 67% identity with human UBA5 at the amino acid level. Similarly, the respective UFM1 and E2 homologues, ubiquitin-like protein and UFC1, share 88% and 79% identity, respectively. Therefore, we used a fly model to determine whether the identified mutations in our patients would impair the function of the UFM1 pathway in the nervous system. As a loss-of-function of UBA5 is associated with ARCA, *FRT UBA5*, and *hs-flp* were crossed to generate *UBA5* null mutant fly lines. Unfortunately, *UBA5* null mutant flies of both sexes died during embryonic development. *UBA5 RNAi* flies were found to exhibit an 80% reduction in *dUBA5* mRNA expression. Here, dUBA5 knockdown was applied to model UBA5-associated ARCA and explore the in vivo functions of the UFM1 system. The resulting flies were analyzed at 3 days of age.

**Fig 3:**
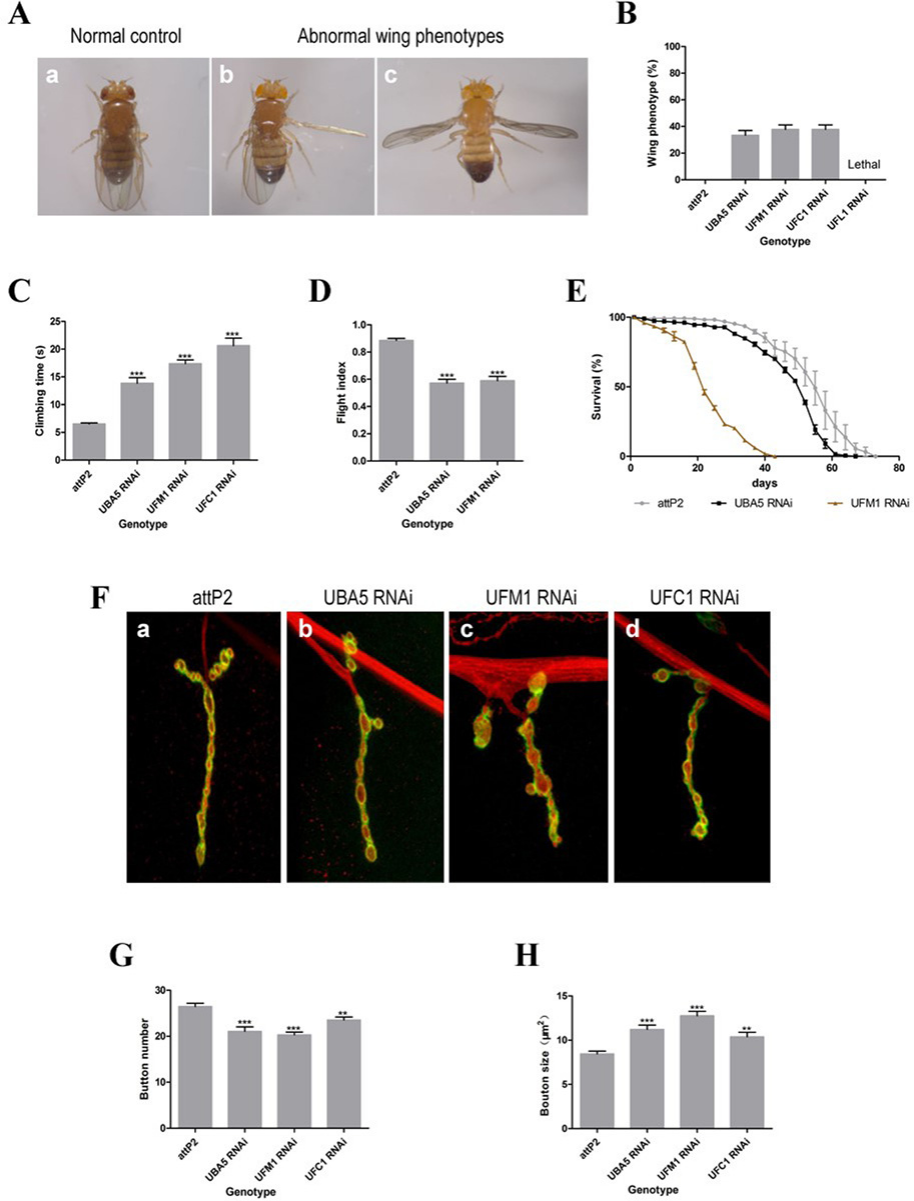
RNAi-mediated knockdown of UBA5 and other molecules of UFM1 pathway induce neurodegeneration. (A) Knockdown of UBA5 and other crucial molecules of UFM1 pathway, UFM1 and UFC1, in Drosophila exhibit abnormal vertical-turned wings that fail to fold, whereas knockdown of UFL1 is fatal. The wing phenotypes are: normal (a), unilateral wing abnormality (b), bilateral wing abnormality (c). (B) Percentage of abnormal wing phenotypes. (C) Comparison of climbing abilities shows climbing disabilities of the RNAi flies. (D) Comparison of flight abilities presents flight declines of the RNAi flies. (E) The life spans of the RNAi flies are significantly shortened. (F) The confocal images of larval muscle exhibit reduced type Ib bouton number and increased bouton size of the RNAi flies. (G) The statistical graph of bouton numbers. (H) The statistical graph of bouton sizes. All the indicated genotypes in panel B-E are: *DA-GAL4>attP2*, *DA-GAL4>UBA5 RNAi*, *DA-GAL4>UFM1 RNAi*, *DA-GAL4>UFC1 RNAi*, *DA-GAL4>UFL1 RNAi*. All the indicated genotypes in panel F-H are: *OK6-GAL4>attP2*, *OK6-GAL4>UBA5 RNAi*, *OK6-GAL4>UFM1 RNAi*, *OK6-GAL4>UFC1 RNAi*. *** = p < 0.0001 and ** = p < 0.*01*, Student’s *t*-test and Mann–Whitney test.

UBA5 knockdown flies (both male and female) exhibited abnormal vertical-turned wings that failed to fold under resting conditions at 25°C. The wing postures indicated unilateral or bilateral wing abnormalities, and the percentages of abnormal wing phenotypes are shown in Fig 3A and 3B. Similar phenotypes were observed in *dUFM1 RNAi* and *dUFC1 RNAi* lines, whereas UFL1 knockdown was found to be lethal. In contrast, the control flies always held their wings parallel to the body axis. The penetrance of this phenotype increased when flies were raised at 29°C; specifically, 56.9% of the *dUBA5 RNAi* lines were found to exhibit wing defects. The climbing and flight abilities and lifespans of *dUFM1 RNAi*, *dUBA5 RNAi*, and *dUFC1 RNAi* lines were greatly reduced, compared with control flies (Fig 3C and 3D). In an evaluation of the climbing abilities of these flies after removing the wings to exclude wing involvement in climbing movements, a similar tendency was observed (data not shown).

A histological analysis revealed no disruption in muscle morphology in the indirect flight muscles of *Da-Gal4 > dUFM1 RNAi* and *Da-Gal4 > dUBA5 RNAi* flies. Defective NMJs remained another anatomical explanation for the observed abnormal wing posture. Confocal images of larval muscles revealed a reduced number of type Ib boutons and increased bouton size in *UFM1 RNAi*, *UBA5 RNAi* and *UFC1 RNAi* flies relative to WT controls; this effect was particularly pronounced in the motor neurons controlled by an *OK6-Gal4* driver (Fig 3E–3H). We expressed *dUFM1 RNAi* and *dUBA5 RNAi* specifically in the nervous system using the *Elav-Gal4* driver and in muscle cells using the *MHC-Gal4* driver. The above-described bouton changes were also observed with neuron-specific *Elav-Gal4*, but not *MHC-Gal4*, indicating that the UFM1 pathway is active in neurons. We noticed that all UFM1 knockdown phenotypes were highly reminiscent of those associated with UBA5, albeit stronger.

### Overexpression of wild-type UBA5 significantly restored neural lesions in *UBA5 RNAi* flies, whereas mutant UBA5 could not confer effective restoration

**Fig 4:**
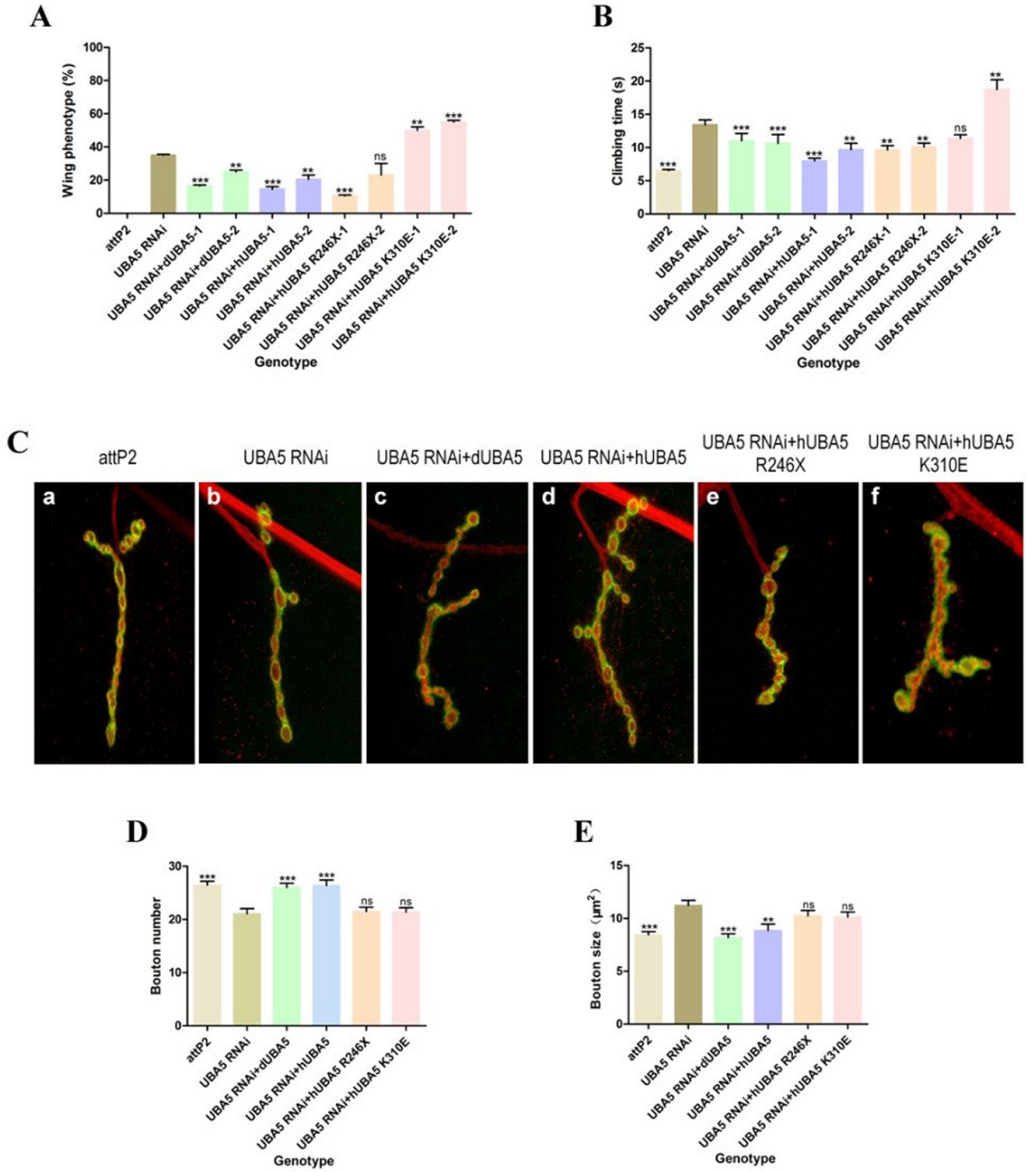
Overexpression of wild-type UBA5 significantly restores neural lesions of UBA5 RNAi flies while mutant UBA5 could not confer effective rescue. (A) Percentage of abnormal wing phenotypes. (B) Comparison of climbing abilities. (C) The confocal images of larval muscle. (D) The statistical graph of bouton numbers. (E) The statistical graph of bouton sizes. All the indicated genotypes in panel A-B are: *DA-GAL4>attP2*, *DA-GAL4>UBA5 RNAi*, *DA-GAL4>UBA5 RNAi+dUBA5-1*, *DA-GAL4>UBA5 RNAi+dUBA5-2*, *DA-GAL4>UBA5 RNAi+hUBA5-1*, *DA-GAL4>UBA5 RNAi+hUBA5-2*, *DA-GAL4>UBA5 RNAi+hUBA5 R246X-1*, *DA-GAL4>UBA5 RNAi+hUBA5 R246X-2*, *DA-GAL4>UBA5 RNAi+hUBA5 K310E-1*, *DA-GAL4>UBA5 RNAi+hUBA5 K310E-2*. All the indicated genotypes in panel C-E are: *OK6-GAL4>attP2*, *OK6-GAL4>UBA5 RNAi*, *OK6-GAL4>UBA5 RNAi+dUBA5-1*, *OK6-GAL4>UBA5 RNAi+hUBA5-1*, *OK6-GAL4>UBA5 RNAi+hUBA5 R246X-1*, *OK6-GAL4>UBA5 RNAi+hUBA5 K310E-1*. *** = p < 0.*0001*, ** = p < 0.*01* and ns = no significance, Student’s *t*-test and Mann–Whitney test.

We generated transgenic fly lines that would overexpress *Drosophila UBA5*, wild-type human *UBA5*, and mutated or deleted human *UBA5* to investigate whether the identified mutations would impair the function of UBA5. Quantitative real-time PCR was performed to confirm similar expression levels. Rescue experiments were performed to determine whether mutated *UBA5* could rescue the neurodegenerative defect caused by the loss of the *Drosophila* ortholog UBA5. The expression of wild-type hUBA5 and dUBA5 in the UBA5 knockdown fly significantly reduced the observed neurodegeneration but was unable to completely convert the phenotype to wild-type (Fig 4). The rescued flies exhibited an increased climbing speed. Consistent with these findings, significant NMJ restoration was observed with UBA5 expression, and the size within these regions was also markedly restored (Fig 4C and 4E). As shown in Fig 4A and 4B, the expression of mutant UBA5 could not rescue the wing phenotype and climbing defect.

## Discussion

ARCAs are genetically heterogeneous. At present, a large proportion of ARCA cases remain unexplained by mutations in known genes. Here we uncovered the genetic basis for a new subtype of ARCA. This report demonstrates that ataxia can be caused by mutations in *UBA5* (NM_198329.2). Both affected individuals carried a mixture of two mutant alleles (c. 568 C > T; p. R246X and c. 760A > G; p. K310E), whereas their parents were heterozygous mutation carriers [the mother (I:2) carried c. 568 C > T (p. R246X) and the father (I:1) carried c.760A > G (p. K310E)]. Both variants were located in the conserved region and cosegregated completely (Fig 1D). Cases with copy number variations in the *UBA5* and *UFM1* region have been documented in the ClinVar database. A 0.4-Mb heterozygous loss in 3q22.1, which includes *UBA5* and *NPHP3*, was previously detected in a patient with global developmental delays, impaired hearing, muscular hyptonia, and seizures. Because *NPHP3* functions in the renal tubular development process, the loss of UBA5 may have resulted in these phenotypes. A 1-Mb duplication in 13q13.3, including *UFM1*, was also identified in a case with global developmental delays, gait disturbance, and other abnormalities. Our molecular characterization of UBA5-R246X revealed a loss in UFM1 activation in the absence of the catalytic cysteine Cys250 (Fig 2). The activity of UBA5-K310E was not clear; the interaction between this mutant protein and UFM1 was maintained but less stable, which may affect its ability to activate UFM1 (Fig 2).

UBA5 is a member of the ubiquitin-activating protein family and the only known E1 enzyme in the UFM1 cascade. In humans, *UBA5* is transcribed as two distinct isoforms (1–404 and 57–404).The role of the additional N-terminal residues encoded by the longer splice transcript is not clear; however, these residues are not strongly conserved and are not required for ubiquitin-like modifier (UBL) activation. [8,14] UBA5 knockout mice were found to die in utero because of severe anemia associated with the defective differentiation of both megakaryocytes and erythrocytes. [17] However, there are no previous reports of a relationship between UBA5 and neurological disorders. The form of ARCA described herein is the new phenotype to be associated with impairment in the ubiquitin-like system. Emerging evidence of mutations in the ubiqutin system have been found in cerebellar ataxia patients, as was described in our report as well as others. [18,19]

UFM1 belongs to the UBL family, the members of which are present in nearly all eukaryotic organisms, with the exception of fungi. As described in detail previously, the UFM1 cascade, which includes the ligase UFL1, UFM1-specific protease UfSP2, and known target proteins (Ufbp1 and Cdk5rap3), has been implicated in ER functioning and cell cycle control. [20,21] A loss-of-function of the UFM1 cascade in mice leads to apoptosis in fetal liver cells and pancreatic beta cells. [13,17] In particular, the ligase UFL1 was shown to be involved in spinocerebellar ataxia type 1 (SCA1), a polyglutamine disease. Furthermore, UFL1 deficiency contributes to SCA1 pathology through a functional deficiency in Bergmann glia, which regulate cell proliferation through the regulation of CDK5RAP3. [22]

*Drosophila* was selected as a model with which to study the involvement of *UBA5* in the disease phenotype. The *Drosophila* larval NMJ is a well-established synaptic model system that shares major features with central excitatory synapses in the mammalian brain and has been successfully used to investigate human neural disorders, as described in detail previously. [23] Our *UBA5* knockdown *Drosophila* models exhibited abnormal wing phenotypes, a shortened life span and climbing disabilities. Neuron-specific *UBA5* knockdown resulted in reduced NMJ growth and a consistent decrease in synaptic size, which presented as a reduced number of type Ib boutons and increased bouton size in the larval muscles, suggesting that the UFM1 cascade might play a role in the development of NMJs in *Drosophila*. Our data thus shed light on a role for the UFM1 cascade in neurological diseases.

In conclusion, we identified mutations in *UBA5*, an E1 enzyme, in patients with ARCA. A *Drosophila* model provided lines of evidence suggesting that a UFM1 pathway impairment might contribute to the neurological phenotypes of ARCA. Rescue experiments involving wild-type and mutated human *UBA5* definitively demonstrated the deleterious nature of these mutations and shed light on the underlying molecular pathogenesis of this disease. An analysis of the UFM1 pathway should be performed in patients presenting with both sporadic and familial ARCA.

## Abbreviations

ARCA: autosomal recessive cerebellar ataxia
UBA5: ubiquitin-like modifier activating enzyme 5
Ufm1: ubiquitin-like modifier
UBLs: ubiquitin like molecules
RT-PCR: Reverse transcription PCR
